# Characterisation of a GII-4 norovirus variant-specific surface-exposed site involved in antibody binding

**DOI:** 10.1186/1743-422X-6-150

**Published:** 2009-09-25

**Authors:** David J Allen, Rob Noad, Dhan Samuel, Jim J Gray, Polly Roy, Miren Iturriza-Gómara

**Affiliations:** 1 Enteric Virus Unit, Virus Reference Department, Centre for Infections, Health Protection Agency, Colindale, London, NW9 5EQ; 2 Pathogen Molecular Biology Unit, Department of Infectious & Tropical Diseases, London School of Hygiene & Tropical Medicine, Keppel Street, London, WC1 5HT; 3 Serology Development Unit, Virus Reference Department, Centre for Infections, Health Protection Agency, Colindale, London, NW9 5EQ; 4 Department of Pathology and Infectious Diseases, Royal Veterinary College, Hawkshead Lane, Hatfield, AL9 7TA, UK

## Abstract

**Background:**

The human noroviruses are a highly diverse group of viruses with a single-stranded RNA genome encoding a single major structural protein (VP1), which has a hypervariable domain (P2 domain) as the most exposed part of the virion. The noroviruses are classified on the basis of nucleotide sequence diversity in the VP1-encoding ORF2 gene, which divides the majority of human noroviruses into two genogroups (GI and GII). GII-4 noroviruses are the major aetiological agent of outbreaks of gastroenteritis around the world. During a winter season the diversity among the GII-4 noroviruses has been shown to fluctuate, driving the appearance of new virus variants in the population. We have previously shown that sequence data and *in silico *modelling experiments suggest there are two surface-exposed sites (site A and site B) in the hypervariable P2 domain. We predict these sites may form a functional variant-specific epitope that evolves under selective pressure from the host immune response and gives rise to antibody escape mutants.

**Results:**

In this paper, we describe the construction of recombinant baculoviruses to express VLPs representing one pre-epidemic and one epidemic variant of GII-4 noroviruses, and the production of monoclonal antibodies against them. We use these novel reagents to provide evidence that site A and site B form a conformational, variant-specific, surface-exposed site on the GII-4 norovirus capsid that is involved in antibody binding.

**Conclusion:**

As predicted by our earlier study, significant amino acid changes at site A and site B give rise to GII-4 norovirus epidemic variants that are antibody escape mutants.

## Background

The ability of RNA viruses to maintain plasticity as well as functionality in their genome has been well documented as a survival mechanism, allowing RNA viruses to adapt to changes in their environment, maintaining fitness in the viral population [1]. Mutation *in vivo *can have a number of effects including increasing the virulence of a virus [2] or acquisition of antiviral resistance [3,4]. An important consequence of the accumulation of point mutations in viral structural proteins is the rise of antibody escape mutants [5-7]. RNA viruses generate this diversity in their genome via the lack of fidelity of the viral RNA-dependent RNA polymerase (RdRp), and the mutants with most increased fitness are selected from the progeny by environmental factors such as the host immune response.

Norovirus is a genus in the *Caliciviridae *family, that includes pathogens of humans and animals [8]. Human noroviruses are a highly diverse group of viruses with a single-stranded RNA genome made up of three open reading frames (ORFs), [9]. Noroviruses are classified on the basis of nucleotide sequence diversity in the ORF2 gene, which divides the majority of human noroviruses into two genogroups (GI and GII) and approximately 19 genetic clusters within them [10]. The genogroup II-genotype 4 (GII-4) noroviruses have been the dominant circulating strain since the early 1990s [11], and in 2002 a variant GII-4 norovirus emerged that caused unusually high numbers of outbreaks of gastroenteritis in the summer of 2002, and epidemic gastroenteritis around the world in the winter of 2002/2003 [12]. This variant possessed a 3 nucleotide (nt) insertion in the hypervariable P2 domain of the VP1 protein at position 6265. This epidemiological pattern was repeated in 2006 when another novel GII-4 norovirus variant emerged, however, no insertions or deletions were observed in the genome of this virus (J Gray, personal communication).

Noroviruses are the major aetiological agent of outbreaks of gastroenteritis in the community and in semi-closed settings around the world. During a winter season (September-March), the diversity among the GII-4 noroviruses has been shown to fluctuate, driving the appearance of new virus variants in the population [13]. Studies of the genetic diversity of these viruses have shown that new GII-4 variants appear periodically in the population following evolution of the viruses along neutral networks, and that accumulation of mutations in the hypervariable P2 domain results in antibody escape mutant viruses which go on to cause epidemic gastroenteritis [14-16].

Computer modelling experiments have previously suggested that there are two 3-amino acid motifs (site A and site B) in the hypervariable P2 domain that define the appearance of epidemiologically significant GII-4 variant norovirus strains [14]. Based on these observations, we predicted that these two motifs may be a functional variant-specific epitope that evolves under selective pressure from the host immune response and give rise to antibody escape mutants.

Due to the lack of a tissue culture system [17] and suitable animal models in which to study noroviruses, we synthesised recombinant virus-like particles (VLPs) using a baculovirus expression system based on previously described methods [18,19]. These VLPs were used to generate monoclonal antibodies (mAbs) in order to test the functionality of site A and site B. We use these novel reagents to provide evidence that site A and site B form a conformational, variant-specific, surface-exposed site on the GII-4 norovirus capsid that is involved in antibody binding, and that as predicted, significant amino acid changes at site A and site B give rise to GII-4 norovirus epidemic variants that represent antibody escape mutants.

## Results

### Construction of Recombinant Baculoviruses Expressing GII-4 Norovirus VLPs

Analysis of sequence data for the strains used in this study showed >88% nucleotide identity in the ORF2 gene between strains GII-4v0, GII-4v2 and the reference strain Lordsdale virus (LV), and >90% nucleotide identity between GII-4v0 and GII-4v2 sequences (data not shown). Deduced amino acid sequences for VP1 revealed >95% similarity between the GII-4v0 virus and LV (data not shown). Detailed comparison of coding and non-coding nucleotide sequence substitutions showed that the majority of non-synonymous substitutions occurred in the P2 domain (data not shown)

**Figure 1:**
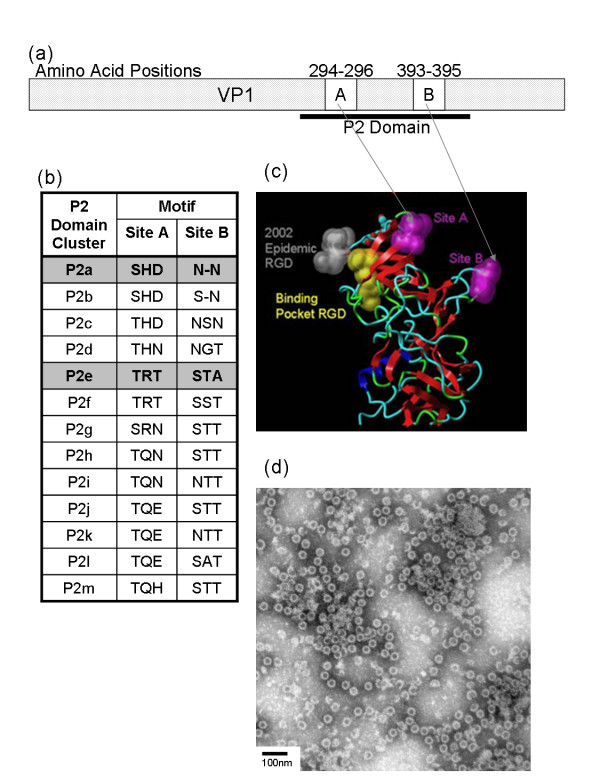
**GII-4 Norovirus Major Sturctural Protein**. (a) Schematic representing the norovirus VP1 protein, highlighting the hypervariable P2 domain and putative epitopes Site A and Site B (as described in Allen *et al.*, 2008). (b) Table showing amino acid variation at Site A and Site B in the period 1999-2006, as previously described in (Allen *et al.*, 2008). Strains used in the work described here are highlighted. (c) Model of the norovirus VP1 P domain showing the location of Site A and Site B in the three-dimensional protein (structure from Cao *et al.*, 2007). (d) Electron micrograph showing GII-4v2 VLPs purified from *Sf*9 cells, the morphology of which is representative for all VLPs described here. Magnification is 105 000× and VLPs are stained with 1.5% phosphotungstic acid. Scale bar is 100 nm.

Wild-type VLPs were purified from recombinant baculovirus-infected *Sf9 *cells and were analysed by EM (Figure 1(d)). Negative staining of preparations showed intact VLPs had formed for both strains, and no significant morphological variation was observed within the VLP preparations (Figure 1(d)). Particles were 30-35 nm in diameter, and the surface structure of the particles could be visualised at high magnification (Figure 1(d)). The VLPs were morphologically indistinguishable from native norovirus virions found in clinical specimens, but >10-fold more VLPs were observed per viewing field than are typically observed in clinical specimens.

**Figure 2:**
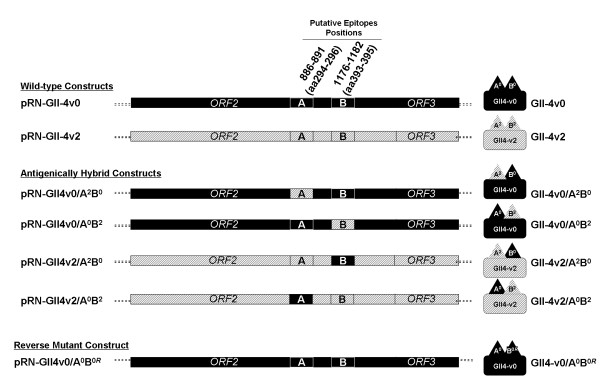
**Schematic representation of norovirus protein coding region of pRN16 constructs expressing wild-type and hybrid VLPs**. Following construction of plasmids pRN-GII4v0 and pRN-GII4v2 expressing wild-type GII-4v0 and GII-4v2 VLPs, respectively, these plasmid constructs were modified by site directed mutagenesis at either putative epitope site A (nt 886-891, aa 296-298), or putative epitope site B (nt 1176-1182, aa 383-395) to generate plasmid constructs that expressed hybrid VLPs. The schematic shows a representation of the region of the plasmid encoding norovirus structural proteins (3'UTR and remainder of plasmid not shown for clarity). PCR mutagenesis was used to generate plasmid constructs that encoded an ORF2 identical to either GII-4v0 or GII-4v2, except at either site A or site B, which was modified to be as equivalent to that position in the heterologous variant. The resulting expressed VP1 protein was a hybrid of the two variants, and so the VLP formed from the hybrid VP1 was antigenically hybrid. Plasmid construct names are given on the left, whilst the hybrid VLPs are represented with VLP names next to them on the right. All GII-4v0 derived regions are shown in solid black, all GII-4v2 derived regions are shown in hatched lines.

The two wild-type expressing plasmid constructs pRN-GII4v0 and pRN-GII4v2 were modified by PCR site directed mutagenesis so that the ORF2 encoded a VP1 protein that was identical to either the GII-4v0 or GII-4v2 parental protein except at either of the putative epitope positions 296-298 (site A) or positions 393-395 (site B), where the protein would be of the heterologous (non-parental) strain (Figure 2). Successful mutagenesis at the target site without alteration of the remaining norovirus insert was confirmed through sequence analysis (data not shown).

All four hybrid VLP-expressing recombinant baculoviruses efficiently expressed VP1 (data not shown). Further, EM analysis of GII4v0/A^2^B^0^, GII4v0/A^0^B^2 ^and GII4v2/A^2^B^0 ^VLPs formed by mutant recombinant VP1 proteins were morphologically indistinguishable from wild-type VLPs (data not shown). However, the hybrid construct pRN-GII4v2/A^0^B^2 ^did not form VLPs, despite expressing VP1 (data not shown), therefore the hybrid VLP GII-4v2/A^0^B^2 ^was not available for subsequent work.

In addition to the hybrid VLPs described above, a reverse mutant construct, pRN-GII4v0/A^0^B^0*R*^, was also generated by back mutation of the hybrid construct pRN-GII4v0/A^0^B^2 ^by site-directed mutagenesis (Figure 2). This reverted the amino acid sequence at the site B from STA (GII-4v2) back to N~N (GII-4v0). This plasmid was sequenced to confirm the mutation at the target site had taken place, and that no changes had taken place in the rest of the insert (data not shown). The plasmid was used as described to produce recombinant baculoviruses and produce the VLP GII-4v0/A^0^B^0*R*^.

### Production and Characterisation of Variant Specific Anti-GII-4 Norovirus Monoclonal Antibodies

Mice were inoculated with either GII-4v0 VLPs or GII-4v2 VLPs, and five monoclonal antibodies were fully characterised for their isotype, titre and binding specificity (Table 1). Test bleeds from both GII-4v0 and GII-4v2 inoculated mice prior to fusion showed that only a low level of cross-reactivity with the heterologous antigen by ELISA (data not shown).

**Table 1:** Monoclonal antibody characterisation.

**Monoclonal Antibody**	**Homologous Antigen**	**Isotype**	**Titre^1^**	**% Reduction in Binding to Urea-Treated Homologous Antigen**
mAbGII4v0.5	GII-4v0	IgG1	100	71.8%

mAbGII4v0.8	GII-4v0	IgG2a	10 000	80.9%

mAbGII4v0.10	GII-4v0	IgG2b	10 000	79.8%

mAbGII4v2.5	GII-4v2	IgG1	10 000	44.0%

mAbGII4v2.6	GII-4v2	IgG1	10 000	51.2%

^1^Titres were determined by serial dilution between 1:10 and 1:10 000 000 in an EIA. Titres were taken as the reciprocal of the last dilution to give an optical density >0.5 at 450 nm.

Three anti--GII-4v0 mAbs (mAbGII4v0.5, mAbGII4v0.8, mAbGII4v0.10) and two anti-GII-4v2 mAbs (mAbGII4v2.5, mAbGII4v2.6) were characterised for reactivity, isotype and titre. The ability of the five mAbs to recognise their respective homologous antigen that had been chemically denatured was also assessed by ELISA.

Urea treatment of the homologous antigen significantly reduced recognition of the epitope by all of the monoclonal antibodies (Table 1), and urea treatment of the heterologous antigen did not confer recognition (data not shown).

### Identification of a Variant-Specific Surface-Exposed Site Involved In Antibody Binding

**Figure 3:**
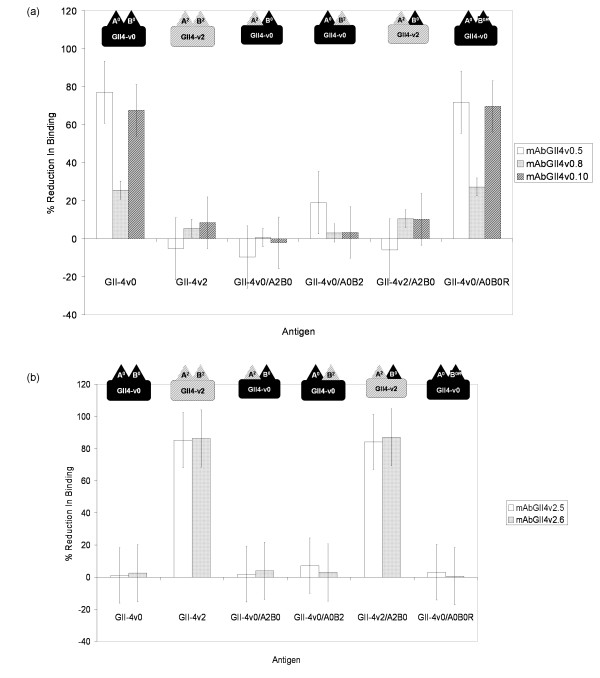
**Average percent reduction in binding of (a) anti-GII-4v0 and (b) anti-GII-4v2 mAbs to wild-type and mutant VLPs in a cross adsorption ELISA**. As described in the Materials and Methods, each mAb was pre-incubated in a blocking step with the antigen indicated on the x-axis, before being transferred to a microtitre plate coated with antigen homologous to the mAbs being tested. Percent reduction in binding was then calculated using a PBS control. Cross absorption assays were repeated 3 times independently and the average data is presented here with bars showing the standard error of the mean. Cartoons representing the antigenic structure of the antigen (as described in Figure 2) are shown above the bars (with corresponding labels below the bars). All mAbs were used at 1:10 000 dilution, except mAbGII4v0.5, which was used at 1:1000.

Each of the three anti-GII-4v0 mAbs displayed a slightly different binding pattern, although the data suggested that all three mAbs recognised an epitope formed or influenced by both site A and site B (Figure 3(a)).

More than 75% reduction in binding of mAbGII4v0.5 to its homologous antigen (GII-4v0) was observed following blocking with the homologous antigen, whereas no reduction in binding was observed following incubation with the heterologous antigen (GII-4v2). Following replacement of GII-4v0 site A with the heterologous GII-4v2 site A, VLP GII-4v0/A^2^B^0 ^failed to block any of the binding of mAbGII4v0.5 to its homologous antigen, demonstrating that site A is essential for mAb recognition of the antigen. When the GII-4v0 site B was replaced with the GII-4v2 site B, VLP GII-4v0/A^0^B^2^, reduced binding to the GII-4v0 antigen by ~19% indicating that when the homologous site A was intact, partial mAb recognition occurred, but without the corresponding homologous site B, mAb recognition was impaired. Similarly, substitution of the heterologous site B in the GII-4v2 with the GII-4v0 site B (VLP GII-4v2/A^2^B^0^) was not sufficient for recognition of this hybrid VLP by mAbGII4v0.5. Importantly, restoring site B to the VLP GII-4v0/A^0^B^2 ^was sufficient to restore wild-type levels of binding, to >70% reduction.

A 25% reduction in binding was observed following blocking of mAbGII4v0.8 with the homologous GII-4v0 VLP, compared to only 5% reduction in binding following blocking with the heterologous GII-4v2 VLP. Blocking with any one of the three hybrid VLPs resulted in ≤10% reduction in binding; the highest level of reduction following blocking among these VLPs was with the GII-4v2/A^2^B^0 ^VLP which displays site B from the homologous (GII-4v0) antigen, thereby indicating that mAbGII4v0.8 was able to partially recognise site B, but that complete recognition required the homologous site A to be displayed concurrently. Blocking with the reverse mutant VLP GII-4/A^0^B^0*R *^restored binding reduction to 27%, comparable to blocking levels by wild-type GII-4v0. This further supports the observation that both homologous site A and site B must be displayed simultaneously on the virus surface for mAb recognition.

Approximately 68% of mAbGII4v0.10 binding was reduced following blocking by GII4v0 VLP, and only one tenth of this reduction in binding observed following blocking with GII-4v2 VLP. Blocking with any one of the three hybrid VLPs resulted in ≤10% reduction in binding; again the highest level of reduction following blocking with a hybrid VLP was with the GII-4v2/A^2^B^0 ^VLP which displays site B from the homologous (GII-4v0) antigen, suggesting a role for site B in mAb recognition. Blocking with the reverse mutant VLP GII-4/A^0^B^0*R *^restored binding reduction to levels comparable to blocking levels by wild-type GII-4v0, of approximately 70% reduction. This confirmed that both homologous site A and site B must be present for mAb recognition of the antigen.

Both anti-GII-4v2 mAbs behaved the same in competition immunoassays (Figure 3(b)). Blocking of both mAbs with the homologous GII-4v2 antigen resulted in >85% reduction in binding in both mAbs, and blocking with the heterologous GII-4v0 antigen resulted in <2.5% reduction in both mAbs. When blocking was performed using one of the two GII-4v0-derived hybrid VLPs (GII-4v0/A^2^B^0^, or, GII-4v0/A^0^B^2^), or the reverse mutant VLP, <7.5% reduction in binding was observed in both anti-GII-4v2 mAbs. In contrast, blocking with the hybrid VLP GII-4v2/A^2^B^0 ^(which is all GII-4v2 except at site B) produced reduction in binding equivalent to that observed with the GII-4v2 antigen of >80% reduction. These data indicated that both the anti-GII-4v2 mAbs recognised an epitope that is variant specific, but is not formed of either site A or site B, alone or in combination.

## Discussion

Efforts to identify sites on the norovirus capsid involved in antibody binding have been hampered by the lack of a cell culture system for human noroviruses [17], and therefore epitope mapping studies using infectious virus have not been possible. Here we have used VLPs synthesised in the baculovirus expression system (BES) as a surrogate for infectious virus in a mutagenesis study to identify sites on the GII-4 norovirus capsid important in antibody recognition. Previous work has shown that when VLPs expressed in the BES were compared with VLPs expressed in a mammalian recombinant protein expression system, no discernable differences in the biochemistry or structure of the two differently expressed VLPs were observed [20].

The data presented here show the expression of high yields of VLPs representing two norovirus strains, one from each of the previously identified neutral networks: (i) pre-2002 epidemic, and (ii) 2002 epidemic-200 [14]. These VLPs were used to immunize mice to produce monoclonal antibodies against these strains.

Both the anti-GII-4v0 and anti-GII-4v2 polyclonal antibody responses were generally specific for the homologous antigen, but a low level of cross-reactivity was observed (data not shown). Cross-reactivity is expected in polyclonal serum because the different antibodies present recognise a range of different epitopes, and have different affinities; therefore polyclonal antibodies will, at least in part, recognise a heterologous antigen. However, following sub-cloning by limiting dilution, cross-reactivity was lost as mAbs were isolated. This confirmed the specificity of these antibodies for a single GII-4 norovirus variant strain through recognition of an epitope that was unique to that variant norovirus and offered no cross-reactivity between other GII-4 norovirus variants.

The absence of any cross-reactivity in the EIA between the mAbs and their heterologous antigen also showed that the mAbs were not recognising epitopes from baculovirus proteins or from *Sf9 *cell-derived proteins. Both antigen preparations were made in the same protein expression system and purified in the same way. Therefore any baculovirus or cell-derived proteins that co-purified with the VLPs were present in both the GII-4v0 and the GII-4v2 VLP preparations used in the immunization of the mice and in the preparation used as antigen in the EIA. Thus any mAb reacting to a baculovirus or cell-derived protein would react equally with both antigen preparations. This is not the case, with all mAbs displaying specificity for the homologous antigen preparation, thereby demonstrating that all five mAbs were raised against norovirus proteins and not baculovirus or insect cell proteins.

Urea treatment of both GII-4v0 and GII-4v2 VLPs revealed the three anti-GII-4v0 mAbs recognised a conformational epitope, whereas the anti-GII-4v2 mAbs recognised a partially conformational epitope (Table 1). Treatment of a macromolecular protein with a chaotropic agent such as 8 M urea will denature the three-dimensional structure of the protein by disrupting the non-covalent intra-molecular interactions such as hydrogen bonding and van der Waals forces. If the mAbs recognised a linear epitope, binding would remain unaffected following chaotropic treatment. However, the epitope recognised by the mAbs must be conformational, as the level of mAb binding to the antigen was reduced following denaturing treatment of the antigen. Demonstrating that the mAbs recognised conformational epitopes was important because site A and site B identified by sequence analysis [14] were shown to be surface exposed loop structures on the virus surface separated by 100 amino acid residues in the linear protein, but in close proximity in the three-dimensional protein (Figure 1(a) &1(c)). Therefore, it was expected that any antibodies raised against these sites would, at least in part, recognise the conformation of the surface structure at these positions, which is why it was important that VLPs were used as the immunogen rather than linear VP1 protein. This was corroborated through the failure to detect a VP1 band in western blots (data not shown).

Site-directed mutagenesis was used to modify the norovirus ORF2 gene in the plasmids pRN-GII4v0 and pRN-GII4v2 at putative epitopes site A (aa296-298) or site B (aa393-395). The aim was to generate both GII-4v0 and GII-4v2 hybrid VLPs which displayed either an heterologous site A or site B. It was predicted that the changes engineered at site A or site B would differently affect the ability of mAbs to recognise the antigen, and so demonstrate the roles of site A and site B as surface-exposed sites involved in antibody binding.

The three hybrid VLPs that were isolated were found to be morphologically indistinguishable from wild-type VLPs as determined by EM, demonstrating that the mutagenesis had no adverse effect on the structural integrity of the VLP. The exception was the hybrid VLP expressed from the recombinant baculovirus BAC-GIIv2/A^0^B^2^, which despite expressing VP1 to high levels, did not form VLPs. As no coding errors were observed in the ORF2 gene, and a high level of protein expression was observed by SDS-PAGE, the lack of VLP formation was not due to truncation of the protein, failure of the baculovirus and expression vector to undergo recombination, or failure of the recombinant baculovirus to express the protein. Therefore, it seems most likely that the mutations engineered in the P2 domain were structurally unfavourable and that they either perturb the correct conformation of the protein or interfered with the subunit-subunit interactions, thus precluding particle formation.

It was predicted that the mAbs raised against the GII-4v0 and the GII-4v2 antigens would recognise a site formed of both site A and site B, or would recognise a site formed of one of these sites alone. This was tested in a cross absorption EIA using wild-type VLPs, hybrid VLPs, the reverse mutant VLP and the five mAbs.

All three anti-GII-4v0 mAbs recognised an antigenic region formed or influenced directly by both site A and site B, as replacement of either of these sites abolished recognition of the GII-4v0 antigen by the mAbs. This observation was supported by the data from the reverse mutant VLP. The GII-4v0/A^0^B^2 ^VLP failed to block binding of the anti-GII-4v0 mAbs to the GII-4v0 antigen, but reverse mutation of site B in this antigen back to GII-4v0 concurrently restored the ability of the antigen to block mAb binding. The conclusion that the anti-GII-4v0 mAbs require both site A and site B for antibody binding reflects predictions made using bioinformatics data [14]. It was noted that epidemiologically significant variant strains appeared in the population following a cluster transition event in which biochemically significant amino acid substitutions (or insertions/deletions) were observed at site A and site B concurrently which itself suggested that both site A and site B are required for defining epidemiologically important strains and allowing GII-4 noroviruses to evade immunity existing in the population.

The back mutation of the hybrid GII-4v0/A^0^B^2 ^at site B created the VLP GII-4v0/A^0^B^0*R *^that had twice undergone site-directed mutagenesis at site B, so that it was structurally and antigenically identical to the wild-type GII-4v0 antigen. This experiment confirmed: (i) that the site-directed mutagenesis process had no effect on the integrity of the antigenic properties of the particle, other than those created by the targeted mutation, and, (ii) that recognition of an unrecognised hybrid antigen by a mAb could be restored by replacement of the mutated site, thus demonstrating that the mutated site was necessary for antibody recognition of the antigen.

The anti-GII-4v2 mAbs recognised only the GII-4v2 VLP and the GII-4v2/A^2^B^0 ^VLP, therefore demonstrating that these mAbs recognise either an epitope that is dependent on site A being in the structural context of the GII-4v2 antigen, but is independent of site B, or, an epitope that is formed of neither site A nor site B. The former is difficult to evaluate because the VLP GII-4v2/A^0^B^2 ^was not available, but the latter could be investigated by construction of a GII-4v2 hybrid VLP with site A and site B from GII-4v0, which if recognised by the mAbs, would demonstrate that the mAbs bind a site that is neither site A nor site B. Conversely, if such a VLP was not recognised, this would demonstrate that site A and site B were important for antibody binding, and more detailed mutagenesis studies where individual amino acid residues in the GII-4v2 VLP were mutated would aid in revealing the residues critical for antibody recognition. Whether the five mAbs described here recognise the same or different epitopes remains to be tested in blocking EIAs.

In this study, we have used several well characterised experimental systems in conjunction with *in silico *models, to identify sites on the GII-4 norovirus capsid that are important in antibody recognition. The use of antigenically hybrid VLPs to study capsid-antibody interactions was used as a surrogate for infectious virus because there is no cell culture system available for these viruses, and our approach of systematic mutation of VLPs led to the identification of two 3aa sites on the surface of the capsid required for antibody binding. Whether the regions identified in this work represent neutralisisng epitopes remains to be investigated, but these investigations remain hampered by the lack of a replicative *in vitro *system or suitable animal model. It would also be interesting to determine whether the mAbs described here could interfere with the ability of VLPs to interact with histo-blood group antigens (HBGAs) when used in a VLP-HBGA binding assay [21].

## Methods

### Clinical Samples

Two faecal specimens were selected from outbreaks that had been characterised by PCR as being caused by a GII-4 norovirus at the Enteric Virus Unit, Centre for Infections, Health Protection Agency, London, UK. The two viruses were: (i) a GII-4 norovirus circulating before the 2002 epidemic, classified as a variant 0 (GII-4v0) virus; and, (ii) a GII-4 norovirus circulating after the 2002 epidemic, and classified as a variant 2 (GII-4v2) virus. Samples were prepared as 10% suspensions in balanced salt solution (Medium 199, Sigma, Dorset, UK) prior to nucleic acid extraction.

### Nucleic Acid Extraction & Reverse Transcription

Total nucleic acid was extracted from a 250 μl aliquot of the 10% faecal suspension using a guanadinium isothiocyanate/silica method as previously described [22]. Extracted nucleic acid was incubated at 42°C for 60 minutes with 50 pmol of poly(T)-TVN primer in Tris-HCl buffer, pH8.3, 5 mM MgCl_2_, 1 mM each dNTP, and 200 U SuperScript^® ^III reverse transcriptase (Invitrogen, Paisley, UK).

### PCR and Amplicon Purification

The genes ORF2 and ORF3 encoding the major structural protein VP1 and the minor structural protein VP2, respectively, and the 3' untranslated region (3'UTR), were amplified by PCR using primers ORF1/2-F1 [14] and TVN-linker. The resulting amplicon 3'ORF1+ORF2+ORF3+3'UTR, was either 2513 bp or 2516 bp in length, depending on the strain. Reactions were performed using High Fidelity PCR System (Roche Diagnostics Ltd, Burgess Hill, UK). PCR amplified amplicons were purified either from solution using Montage^® ^PCR Filter Units (Millipore, Watford, UK), or from agarose gels using Geneclean^® ^Spin Kit (Qbiogene, Cambridge, UK). Both were used as according to manufacturers' instructions.

### Amplicon Sequencing and Sequence Analysis

Sequencing PCR was performed using 10 pmol of primer and 100 fmol template DNA. All sequencing was performed using GenomeLab™ DTCS - Quick Start Kit (Beckman Coulter, High Wycombe, UK) according to the manufacturer's instructions, and a CEQ8000 automated sequencer (Beckman Coulter).

Nucleotide sequence contigs were generated from trace data using the Assembler tool in BioNumerics v3.5 (Applied Maths, Kortrijk, Belgium). Multiple alignment and phylogenetic analysis was performed using appropriate algorithms in BioNumerics v3.5 (Applied Maths). Amino acid sequence data was deduced from nucleotide data and analysed using BioEdit [23], and also using BioNumerics v3.5 (Applied Maths).

### Cloning

Each of the purified 3'ORF1+ORF2+ORF3+3'UTR amplicons was cloned into the vector pCR2.1-TOPO^® ^(Invitrogen) according to the manufacturer's instruction. The 3'ORF1+ORF2+ORF3+3'UTR amplicon was then modified by PCR using primers deigned to: (i) remove the partial 3'ORF1 sequence at the 5' end of the amplicon; (ii) include two restriction enzyme sites at each end of the amplicons in order to allow for directional cloning into vector pRN16; and, (iii) modify the translation initiation context of ORF2 to match that of the baculovirus polyhedrin gene (PH). The GII-4v0 amplicon was modified as follows: 5'-A-*Stu*I-*Sac*I-PH-ORF2-ORF3-3'UTR-*Xba*I-*Stu*I-A-3'. The GII-4v2 amplicon was modified as follows: 5'-A-*Stu*I-*Kpn*I-PH-ORF2-ORF3-3'UTR-*Xba*I-*Stu*I-A-3'. The vector pRN16 contains a region of the *Autographa californica *nuclear polyhedrosis virus (AcMNPV) around the polyhedrin gene (ORF7 (735)) which overlaps the essential ORF8 (1629) gene. pRN16 was produced by ligating the *Bst*XI/*Hin*dIII polyhedrin promoter-polylinker fragment from pAcCL29.1 [24] into *Bst*XI/*Hin*dIII cut pBacPAK8 (BD Clontech). After digestion of both inserts and vector pRN16 with the appropriate restriction enzymes ligation was performed using T4 DNA ligase (Fermentas, York) to produce the plasmids pRN-GII4v0 and pRN-GII4v2.

Positive clones were grown overnight in a 50 ml LB broth culture containing 50 μg/ml ampicillin, and the plasmid isolated using a plasmid preparation kit (Plasmid Midi Kit, QIAGEN, West Sussex, or SNAP Midi-Prep Kit, Invitrogen) according to the manufacturer's instructions.

### Site-Directed Mutagenesis

Wild-type sequences for GII-4v0 and GII-4v2 VLPs were mutated in the P2 domain at site previously identified as forming a putative epitope [14] (Figure 1(a)-(c)). Plasmids pRN-GII4v0 and pRN-GII4v2 were mutated in a site specific mutagenic PCR reaction at either a 9 nt site at positions 886-894 (site A), or a 6 or 9 nt site (depending on the strain) at positions 1176-1182 (site B) from the GII-4v0 sequence to the GII-4v2 sequence, or vice versa (Figure 2). For this, the GeneTailor Site-Directed Mutagenesis System (Invitrogen) was used according to manufacturer's instruction, using a touchdown PCR method to mutate and amplify the plasmids. Mutated plasmids were transformed into DH5αT1^R ^*E. coli *cells (Invitrogen) and purified using SNAP Midi Prep Kit (Invitrogen) according to manufacturer's instruction.

Purified plasmids were used to generate recombinant baculoviruses as has been previously described [19]. Following recombination, a clonal population of recombinant baculoviruses was obtained by plaque purification. The resulting recombinant baculoviruses expressed either GII-4v0 VLPs (BAC-GIIv0) or GII-4v2 VLPs (BAC-GIIv2). Plaque purified viruses were used to seed stock cultures of each virus, and these stocks were titred by plaque assay.

### Generation of VLPs

Suspension cultures of *Sf*9 cells were infected with either BAC-GII4v0 or BAC-GII4v2 at a moi of 2-3 and incubated at 28°C for 48-72 hours. Virus-like particles were purified from the intracellular phase by treatment with phosphate buffer containing 1% IGEPAL (Sigma Aldrich) and sequential centrifugation steps for clarification, and finally through 15%-60% sucrose cushions to concentrate the VLPs. Fractions were collected and analysed by SDS-PAGE on a 12% polyacrylamide gel (NuPAGE kit (Invitrogen), according to manufacturer's instruction) and electron microscopy (EM).

### Monoclonal Antibody (mAb) Production

BALB/c mice were inoculated subcutaneously with 100 μg of either wild-type GII-4v0 or wild-type GII-4v2 VLPs in Freunds incomplete adjuvant in order to produce mAbs. After boosting the mice fortnightly on a further four occasions, the spleen cells were harvested and fused with mouse myeloma cells (NSI) by standard procedures [25].

### Hybridoma Cloning, Screening and Selection

The fused cells were dispensed into 96 well tissue culture plates and cultured in RPMI1640+GlutaMAX media (Invitrogen), supplemented with 2% hypoxanthine-thymidine (HT) (Invitrogen), 1% oxaloacetate-pyruvate-insulin (OPI) (Sigma) and 1% antibiotic-antimycotic (AbAm) (Invitrogen). Ten to 14 days post fusion, supernatants from the fusions were tested for antibodies to GII-4v0 and GII-4v2 by EIA as described below. Hybridomas secreting norovirus variant-specific antibodies were then cloned twice by limiting dilution.

### Enzyme-Linked Immunoassay (EIA)

Microtiter plates (Greiner Bio-One, Stonehouse) were coated with either GII-4v0 or GII-4v2 VLPs at a concentration of 1 μg/ml diluted in PBS + 0.08% azide at 4°C. A 100 μl aliquot of test supernatants were diluted between 1 in 100 and 1 in 10000000 in PBST, and detection was performed using a rabbit anti-Mouse IgG-HRP conjugate antibody (Dako, Cambridgeshire) at 1 in 4000 dilution in conjugate diluent (Microimmune) and TMB Substrate (Europa Bioproducts, Cambridge).

### Isotyping of Monoclonal Antibodies

A 100 μl sample of culture supernatant from each hybridoma was added to coated microtiter plates and antibody isotype determined using a goat anti-mouse IgG1a, IgG2a, IgG2b, IgG2c, IgG3 or IgM (Jackson Laboratories, Maine, USA) antibody, diluted 1 in 2000 in conjugate diluent (Microimmune). Detection was performed using rabbit anti-goat HRP-conjugate diluted 1 in 20000 in conjugate diluent (Microimmune) containing mouse serum (Sigma-Aldrich, Dorset, UK) and TMB Substrate (Europa Bioproducts).

### Reactivity of Monoclonal Antibodies with Denatured Antigen

The EIA was performed as described above, but before the addition of the mAb to the plate, the VLP antigen bound to the plate surface was treated with either 8 M urea in PBS or PBS for 1 hour at room temperature. Wells were then washed 3 times with PBST and the EIA performed as described above.

### Competitive Immunoassay

In the competitive immunoassay, monoclonal antibodies were diluted in PBS 1 in 1000 - 1 in 10000 and pre-incubated with either the homologous or heterologous wild-type VLP at a concentration of 1 μg/ml, one of the antigenically hybrid VLPs at 1 μg/ml, or PBST as a control. Pre-incubated monoclonal antibodies were then added to microtiter plates coated with 1 μg/ml of the homologous antigen (as described above) to which the monoclonal antibody was raised. The monoclonal antibody was then allowed to attach, and detected with an anti-mouse HRP conjugate antibody in an EIA as described above. Results are shown as per cent reduction in binding of mAb to homologous antigen (OD_test_) compared to level of binding in PBST control (OD_PBST_): % reduction in binding = ([OD_PBST _- OD_test_]/OD_PBST_) × 100.

## Competing interests

The authors declare that they have no competing interests.

## Authors' contributions

DJA participated in the design of the experiments, conducted the experiments, and drafted the manuscript. RN participated in the design of the experiments, provided reagents and expertise for production of the VLPs, and editing of the manuscript. DS participated in the design of the experiments, provided reagents and expertise for production of the monoclonal antibodies, and editing of the manuscript. JJG participated in the design and coordination of the study, analysis of the data and editing of the manuscript. PR provided reagents, participated in the coordination of the study and editing of the manuscript. MIG participated in the design and coordination of the study, analysis of the data and drafting and editing of the manuscript. All authors read and approved the final manuscript.
